# Differential diagnosis of vergence and saccade disorders in dyslexia

**DOI:** 10.1038/s41598-020-79089-1

**Published:** 2020-12-17

**Authors:** Lindsey M. Ward, Zoï Kapoula

**Affiliations:** IRIS Lab, Neurophysiology of Binocular Motor Control and Vision, CNRS FRE2022 Neurosciences, UFR Biomedical, University of Paris, 45 rue des Saints Pères, 75006 Paris, France

**Keywords:** Saccades, Oculomotor system, Visual system, Paediatric research

## Abstract

Previous studies suggest vergence and saccade abnormalities in dyslexic adolescents. However, these studies are mainly clinically based and do not provide objective measurements of eye movements, but rather subjectively evaluate vergence using haplosopic conditions in which the two eyes are dissociated (via polarizers, prisms, or intermittent spectacles). Other studies have identified deficits with binocular coordination during reading in dyslexics. Yet, there are few studies that provide objective measurements of eye movements in the dyslexic population to help provide more information regarding if these deficits could be due to an intrinsic motor problem or if they are the consequence of poor reading. 47 dyslexic adolescents (18 female, 29 male; mean age 15.5) and 44 non-dyslexic adolescents (22 female, 22 male; mean age 14.8) wore a head-based eye tracker (PupilCore, Pupil Labs, Berlin) which recorded wide angle saccade and vergence eye movements at 200 Hz. Tests were run using the REMOBI device, which produced a saccade or vergence audiovisual target. Analysis of eye movements was performed with lab-developed software, AIDEAL. The results showed statistically significant abnormalities in vergence and saccades. In vergence, dyslexics displayed a reduced amplitude of the visually driven portion of convergence and a longer duration in the initial phase of divergence. In saccades, dyslexic adolescents demonstrated slower saccades in both directions. They also had an increased disconjugate drift in the first 80 or 160 ms following saccades to the right, suggesting poor binocular coordination. For both vergence and saccades, the peak velocity and time to peak velocity was higher and earlier, respectively, in non-dyslexics compared to dyslexics; yet the average velocity of both movements was lower in dyslexics. Thus, these results indicate peculiar velocity profiles in dyslexics, particularly a slow deceleration phase in both vergence and saccades. The study provides an objective method to diagnose vergence and saccade abnormalities while viewing targets in the real three-dimensional space in a dyslexic population. Vergence abnormalities are demonstrated to be a problem in dyslexics, occurring independently from reading. We hypothesize these disconjugate drifts following saccades are the result of slow vergence capacity. Rehabilitation programs, such as those using REMOBI, should aim to target these deficits in vergence velocity, as this has been shown to improve binocular control.

## Introduction

Dyslexia is a developmental learning disorder that emerges in childhood. The primary deficit involves problems with word recognition, decoding words, and spelling, which translates into slower reading, decreased comprehension, and even difficulty writing^1,2^. Though it is a prevalent condition, it is often difficult to describe a clear pathologic presentation of the disorder. Traditionally, dyslexia has been viewed as a primary problem related to a basic phonological processing deficit^3,4^. However, this is not necessarily the case: other underlying deficits have also been found, for example, related to verbal short-term memory and/or rapid automatized naming; though these have been associated with dyslexia, these deficits are not required to satisfy a dyslexia diagnosis^5^. Usually, dyslexia is described as a deficit that manifests itself as a learning disability at the behavioral level^6^. These issues are thought to manifest themselves at the word level, which in turn impacts larger learning issues such as written expression and reading comprehension^4^.

Given its traditional classification as a learning disability, dyslexics often try to compensate for the reading and writing manifestations of their disorder by participating in specific remediation and interventions targeted towards their learning deficits^7^. Dyslexics may also try coping strategies in order to teach themselves ways to interact with their schoolwork (which traditionally focuses on using words, reading, and writing as primary methods for pedagogy) in ways they can process. Finally, with the advent of computers and technology, some dyslexics find it easier to use digital tools in order to compensate, after trying traditional interventions to augment their poor reading and/or spelling skills^8,9^.

Though traditionally dyslexia has been described as a behavioral issue, there have also been studies that have shown that dyslexics do have physiologic deficits as well: they demonstrate evidence of abnormal visual processing, including abnormal saccadic and vergence eye movements, and visual rehabilitation has been recommended for decades to improve symptoms in dyslexic adolescents^1,10–15^. Though dyslexia is considered a learning disability rooted in phonologic processing deficits, and though studies have shown physiologic problems in coordinating eye movements, there has been no longitudinal or randomized interventional trial that has demonstrated whether these physiologic problems are the consequence of poor reading and spell problems, or the cause of these issues. Though this issue of causality or consequence has not been adequately addressed, this study adds to the literature surrounding the characterization of abnormal dyslexic eye movements, particularly with respect to binocular coordination.

### Previous studies on eye movements in dyslexic children

There already exists a vast literature on vergence abnormalities in dyslexia. Buzzelli described an experiment in 1991 in which he measured 13 dyslexic and 13 non-dyslexic stereopsis, accommodative, and vergence facility. He found that dyslexics performed significantly worse than the matched healthy subjects on a test of vergence facility, whereas visual acuity and stereopsis were similar^16^. He concluded that decreased vergence ability may contribute to reading impairment. Other studies are consistent with this finding^17,18^.

An important caveat to the majority of these studies is the way these eye movements are evaluated. Typically, eye movements are indirectly evaluated using optometric or orthoptic methods, which present a number of challenges. First, they are based on clinical, indirect evaluation with an inherently subjective way of measuring. Orthoptic evaluation of vergence rely on prisms, which dissociates binocular coordination and therefore do not provide a measurement of how one sees in real, everyday three-dimensional life. Further, these studies necessitate a subjective component (i.e., asking subjects at which point their vision becomes doubled). For adolescents, this is particularly problematic, as they are more likely to give a subjective and therefore less reliable answer. It is important to note these previously mentioned studies subjectively evaluated eye movement with a mix of clinical and orthoptic measurements, and effective objective measurements of eye movements are scarce. Indeed, our lab has previously demonstrated the importance of using objective measurements to evaluate vergence and accommodation directly, under normal binocular viewing conditions^19^. The need for objective evaluation of eye movements under normal viewing conditions extends to the dyslexic population as well.

More recently, eye movement abnormalities in dyslexic adolescents have been demonstrated using more objective measurements, some resulting in increased latencies of both vergence and saccadic movements^11,17,20,21^. There has even been evidence to show that vergence training may improve reading in dyslexic adolescents (increased number of words read per minute), indicating there is a basis of vergence abnormality to their primary deficit^22^. However, these studies have been conducted on only a small sample size, some of them using targets that cover only a small range of movement, which does not replicate how dyslexics would view larger targets in real life. In addition, there is conflicting evidence for these conclusions in the literature: a study performed in 86 Swedish nine-year old dyslexic children and matched control children did not demonstrate any differences in terms of strabismus, accommodation, stereo acuity, vergence function or ocular dominance using orthoptic and qualitative measurements^23^. Other studies have refuted these findings as well^24^. Finally, previous literature objectively measuring eye movements in dyslexics, including those from our lab, has been studied using visually driven cues. There is evidence that a sound presented simultaneously to a visual target reduces latency, as it acts as a warning stimulus, suggesting visually driven cues alone are not adequate to simulate natural eye movements^25^.

### Reading deficits in dyslexics

How do these contradictory studies relate to reading, the primary outcome that interests dyslexics? This, too, is an area of debate in the literature. Some have found increased vergence and accommodative deficits in dyslexic vs non-dyslexic children while reading^1^. Others have found increased difficulty in binocular coordination, of saccadic movement in dyslexic versus non-dyslexic children^12^. The disconjugacy during the saccadic movement, or the “change vergence”, is hypothesized to result from poor vergence that is normally activated during the saccadic movement to correct the saccade asymmetry between the two eyes^13,26^. In a subsequent study, it was found that binocular yoking of reading saccades was poor in dyslexic children relative to non-dyslexics, resulting in vergence errors during reading. There was considerable variability of the vergence angle from fixation to fixation, creating a larger fixation disparity for dyslexic children, particularly when reading at near distance^13^. This binocular disparity could be related to vergence dysfunction; however at this stage there is no objective study of vergence in dyslexics in the three-dimensional space which would allow us to better understand the relationship between vergence and reading. It would be useful to determine if this change in vergence abnormality exists outside of the reading context in saccadic movements to larger, audiovisual targets, in order to determine if it affects other ways dyslexic children perceive and interact in the world.

Why is it important to relate binocular coordination deficits to reading problems in this specific dyslexic population? Coordinated eye movements that result in good vergence control are necessary in order to obtain a clear single vision of the letters in three dimensional space that compose words, necessary for reading^13^. Indeed, previous studies have shown that when a mismatch of vergence and accommodation is induced experimentally (with the use of prisms or spherical lenses), saccades become more disconjugate (divergent), and the correlation between intra-saccadic disconjugacy and the post-saccadic disconjugacy drift weakens. Therefore, residual disparities during fixation occur, which would interfere with cognition and reading tested with the Stroop test^27^. Saccades themselves are necessary to move the eyes from one word to the next across a line of text. A previous study showed that students who demonstrated vergence problems also demonstrated difficulty in binocular coordination of saccades and lower reading scores. After correcting these vergence abnormalities, the binocular coordination of saccades improved and reading improved (in terms of decreased fixation duration and a decrease in the number of regressive saccades)^28^.

Could the act of reading itself help train binocular coordination, or are these deficits in binocular coordination intrinsic in dyslexics, leading to trouble reading? It is important to note that eye movement deficits have been demonstrated in dyslexics both during and outside of reading. Physiologically, the process of binocular coordination that results in coordinated saccades and vergence is slow and takes most of childhood to develop^29–31^. It is unclear if these abnormal eye movements are a disturbing factor that causes reading deficits, or if these deficits are the result of impaired reading, which could help retrain the eyes and improve coordination of these movements. Either way, this motor control learning in dyslexics is not achieved in the same way as healthy controls, in that binocular coordination is poorer during reading as compared to healthy controls. It may be in some cases that the fundamental reading problem is based in abnormal eye movements, or at least exacerbated by their reading deficits, though there have been no randomized control trials or longitudinal studies to suggest either hypothesis. Therefore, we would consider that this problem with binocular coordination could, at the least, interfere with reading and learning.

### The present study

To date, many clinical studies have suggested vergence and accommodation difficulties in dyslexics without objective evaluation^16–18,22^. Other studies suggest eye movement deficits during reading in dyslexics, including larger amplitudes, longer fixations, frequent regressive saccades, problems coordinating movement to the next line, and poor binocular coordination during saccades and fixations^10–13,32^. Most authors attribute abnormal eye movement in reading to reading difficulty. We suggest an alternative explanation: that these deficits are due to poor binocular motor control, particularly in vergence, that occurs outside of reading.

Therefore, the purpose of this study was two-fold: (1) To objectively evaluate vergence eye movements in dyslexic and non-dyslexic adolescents in three-dimensional space without dissociation of vergence and accommodation; (2) to objectively evaluate saccades in dyslexic and non-dyslexic adolescents, with a particular emphasis on the binocular coordination during and after the saccade, which relies on vergence capability. Given the previous literature, we predicted that dyslexics would objectively demonstrate deficits in both, binocular coordination of saccades and vergence; we hope the specifics of these abnormalities would better inform how these difficulties could be related to their reading disability.

## Results

### Participant’s characteristics

Dyslexic adolescents reported spending more hours on the computer per week than non-dyslexic adolescents (30.8 h vs 13.5 h, p = 0.000). They also reported liking to read less (4.8 vs 6.6, p = 0.01) and liking to go to theaters and films more than non-dyslexic adolescents (8.1 vs 7.2, p = 0.03) on a scale of 1 to 10, where 1 indicated “not at all” and ten indicated “I love it”.

### Clinical visual examination

Dyslexic adolescents had a lower score on the stereoscopic vision test (57.5 arc-seconds vs 25.7 arc-seconds, p < 0.001), indicating difficulty in perceiving depth with very high spatial resolution. They also exhibited higher scores on the Convergence Insufficiency Symptom Survey (24.4 vs 17.7, p = 0.008), indicating symptomatic vergence disorders. A score greater than 21 indicates a symptomatic vergence disorder^33^. The Convergence Insufficiency Treatment Trial Group (CITTG) developed the Convergence Insufficiency Survey in order to create a validated method to quantify and monitor convergence insufficiency symptoms^34^. While traditionally the cut-off score has been 16 in pediatric and adolescent populations, more recent studies have questioned this value and, given the variability adolescents have shown in their answers, some have utilized the adult cut-off of greater than 21^35–37^. Due to these discrepancies, this study chose to use the higher, adult cut-off to indicate convergence disorders.

### Eye movement results

#### Vergence in dyslexics vs non-dyslexics

In terms of the initial phasic component of vergence (the time when velocity exceeds or drops below 5°/s), dyslexic adolescents exhibited a longer duration for divergence (42.6 ms, SD 21.0 ms vs 29.6 ms, SD 9.9 ms; p < 0.00). They also exhibited an increased peak velocity in both divergence and convergence, meaning their speed during the initial acceleration component of the vergence movement was higher than non-dyslexics (Divergence: 90.7°/s, SD 60.3°/s vs 62.0°/s, SD 76.6°/s; p < 0.00; Convergence: 99.3°/s, SD 63.5°/s vs 70.8°/s, SD 42.7°/s; p < 0.00). Yet, dyslexics displayed a decreased average velocity during this initial phasic component in both divergence and convergence (Divergence: 32.7°/s, SD 11.2°/s vs 41.3°/s, SD 36.9°/s; p < 0.00; Convergence: 35.3°/s, SD 14.2°/s vs 46.0°/s, SD 15.8°/s; p < 0.00). Therefore, despite demonstrating a higher peak velocity during the acceleration phase, the subsequent deceleration phase was significantly slower. When examining the total average velocity (the initial phasic component plus the subsequent 160 ms), dyslexics displayed a significantly lower total average velocity for convergence (0.02°/s, SD 0.007°/s vs 0.2°/s, SD 0.008°/s, p = 0.03), but not during divergence (0.01°/s, SD 0.005°/s vs 0.02°/s, SD 0.005°/s; p = 0.3).

In terms of amplitude in the initial phasic component of vergence, there was no significant difference between dyslexics and non-dyslexics (Divergence: 1.5°, SD 1.0° vs 1.2°, SD 0.7°; p = 0.3; Convergence: 2.2°, SD 1.5° vs 2.2°, SD 1.34°; p = 0.5). Yet, the later phases of vergence measured during the subsequent 80 and 160 ms were both significantly smaller. For convergence only, dyslexic adolescents exhibited decreased amplitude in both the 80 and 160 ms phases of movement (80 ms: 0.9°, SD 0.4° vs 1.1°, SD 0.5°, p < 0.00; 160 ms 1.5°, SD 0.7° vs 2.0°, SD 0.9°, p = 0.01), and divergence movements also trended towards decreased values for the dyslexic population (Table 1).

**Table 1:** Means and standard deviations of vergence parameters in dyslexic and non-dyslexic adolescents.

	Dyslexic		Non-dyslexic		p-value
	Average	SD	Average	SD	
Divergence amplitude (°)	1.5	1.0	1.2	0.7	0.25
Convergence amplitude (°)	2.2	1.5	2.2	1.4	0.53
Divergence total amplitude (°)	2.8	1.1	2.8	1.1	0.71
Convergence total amplitude (°)	3.7	1.9	4.1	1.8	0.23
Divergence latency (ms)	334.0	62.5	334.3	69.2	0.96
Convergence latency (ms)	336.5	68.2	314.3	75.1	0.21
Divergence duration (ms)	**42.6**	**21.0**	**29.6**	**9.9**	**0.00**
Convergence duration (ms)	**60.7**	**31.0**	**49.5**	**24.4**	**0.09**
Divergence peak velocity (°/s)	**90.7**	**60.3**	**62.0**	**76.6**	**0.00**
Convergence peak velocity (°/s)	**99.3**	**63.5**	**70.8**	**42.7**	**0.00**
Divergence average velocity (°/s)	**32.7**	**11.2**	**41.3**	**36.9**	**0.00**
Convergence average velocity (°/s)	**35.3**	**14.2**	**46.0**	**15.8**	**0.00**
Divergence total velocity (°/s)	13.4	4.5	14.5	5.1	0.29
Convergence total velocity (°/s)	**16.2**	**6.7**	**19.4**	**7.7**	**0.03**
Fixation disconjugacy 80 ms after divergence (°)	0.7	0.3	0.9	0.8	0.06
Fixation disconjugacy 80 ms after convergence (°)	**0.8**	**0.4**	**1.1**	**0.5**	**0.00**
Fixation disconjugacy 160 ms after divergence (°)	1.3	0.6	1.6	0.7	0.06
Fixation disconjugacy 160 ms after convergence (°)	**1.5**	**0.7**	**2.0**	**0.9**	**0.01**

Variables with significant differences between the two populations (p < 0.05) are bolded.

In terms of total amplitude (adding the initial phase and the amplitude over the subsequent 160 ms), there was no statistically significant difference between the two populations (Divergence: 2.8°, SD 1.1° vs 2.8°, SD 1.1°; p = 0.7; Convergence: 3.7°, SD 1.9° vs 4.1°, SD 1.8°; p = 0.2).

In summary, careful analysis of the vergence trajectory over different periods reveals abnormal vergence execution in dyslexics, e.g. a higher peak velocity but abnormal slowing of the velocity during the deceleration phase. Amplitude analysis reveals decreased values during the later phases of convergence (80 and 160 ms).

### Saccades in dyslexics vs nondyslexics

There was also a significant difference in several parameters in saccades between dyslexic and non-dyslexic adolescents. For saccades either to the left or to the right, during the initial phase of the movement dyslexic adolescents displayed an increased duration, meaning it took them longer to reach the target (Left: 71.0 ms, SD 14.1 ms vs 64.3 ms, SD 11.6 ms; p < 0.00; Right: 68.9 ms, SD 12.0 ms vs 63.2 ms, SD 8.6 ms; p < 0.00). Similar to vergence, they also exhibited an increased peak velocity during the initial phase of the movement (Left: 387.9°/s, SD 160.0°/s vs 280.0°/s, SD 95.8°/s; p < 0.00; Right: 365.1°/s, SD 159.2°/s vs 277.2°/s, SD 97.0°/s; p < 0.00) but a decreased average velocity (Left: 200.6°/s, SD 75.2°/s vs 259.8°/s, SD 61.1°/s; p < 0.00; Right: 197.2°/s, SD 80.0°/s vs 252.7°/s, SD 60.4°/s; p < 0.00), meaning that, like in vergence, their initial movement towards the target is faster than normal controls, but the subsequent deceleration phase is slowed substantially.

To evaluate binocular coordination during fixation after the saccade, we measured by the disconjugacy of the drifts during the first 80 and 160 ms following the end of the saccade, as these two periods were chosen to represent the two time constants of the ocular movement: the extraocular muscle movement and the stabilization of the eye after the saccade^38^. For saccades to the right only, dyslexics had an increased disconjugacy during both phases of fixation (80 ms: 0.9°, SD 0.9° vs 0.6°, SD 0.4°, p = 0.03; 160 ms: 1.2°, SD 1.07° vs 0.8°, SD 0.8°, p = 0.02). Dyslexics displayed an increased disconjugate drift in both phases of fixation for saccades to the left but this observation did not reach statistical significance (see Table 2).

**Table 2:** Saccadic parameters in dyslexic and non-dyslexic adolescents.

	Dyslexic		Non-dyslexic		p-Value
	Average	SD	Average	SD	
Left amplitude (°)	16.8	3.4	16.7	2.0	0.64
Right amplitude (°)	16.2	1.5	16.8	2.0	0.22
Left total amplitude (°)	17.1	1.6	17.4	2.1	0.61
Right total amplitude (°)	16.9	1.4	17.6	2.2	0.25
Left latency (ms)	250.1	49.1	244.7	53.8	0.12
Right latency (ms)	273.3	65.6	265.6	63.1	0.36
Left duration (ms)	**71.0**	**14.1**	**64.3**	**11.6**	**0.00**
Right duration (ms)	**68.9**	**12.0**	**63.1**	**8.6**	**0.00**
Left peak velocity (°/s)	**387.9**	**160.0**	**280.1**	**95.8**	**0.00**
Right peak velocity (°/s)	**365.1**	**159.2**	**277.2**	**97.0**	**0.00**
Left average velocity (°/s)	**200.6**	**75.2**	**259.8**	**61.1**	**0.00**
Right average velocity (°/s)	**197.2**	**80.0**	**252.7**	**60.4**	**0.00**
Left total velocity (°/s)	72.1	14.8	78.2	9.6	0.09
Right total velocity (°/s)	**74.1**	**7.3**	**78.9**	**9.5**	**0.04**
Left fixation disconjugacy 80 ms after saccade (°)	**0.8**	**0.5**	**0.6**	**0.4**	**0.07**
Right fixation disconjugacy 80 ms after saccade (°)	**1.0**	**1.0**	**0.6**	**0.4**	**0.03**
Left fixation disconjugacy 160 ms after saccade (°)	1.0	0.6	0.7	0.5	0.08
Right fixation disconjugacy 160 ms after saccade (°)	**1.2**	**1.1**	**0.8**	**0.4**	**0.02**
Left disconjugacy during saccade (°)	2.9	1.6	2.8	1.5	0.97
Right disconjugacy during saccade (°)	2.5	1.8	2.3	1.2	0.90

Variables with significant differences between the two populations (p < 0.05) are bolded.

The binocular coordination during the saccade (i.e. the amplitude difference between left and right eye) was not significantly different between the two groups (see Table 2).

To determine the relationship between vergence and saccades, we examined the cross-correlation between average velocity and the disconjugate drift after the saccade at 80 ms and 160 ms. For all adolescents, (dyslexic and non-dyslexic) we found the average velocity during vergence is significantly negatively correlated with the disconjugate drift following saccades to the left in the first 80 ms (convergence: r = − 0.2; p = 0.04; divergence: r = − 0.3; p = 0.007) and in the 160 ms following the saccade (convergence: r = − 0.3; p = 0.002; divergence: r = − 0.4; p = 0.000). Therefore, the lower the velocity was during vergence, the higher the deconjugate drift of the saccades.

When looking at dyslexic and non-dyslexic groups individually, a significant negative correlation between average velocity and the disconjugate drift in the following 160 ms after the saccade was found for dyslexics only during convergence following left saccades (r = − 0.36; p = 0.03). Correlations for drifts following rightward saccades did not reach significance. Correlations for healthy children only did not reach significance. For a summary of these correlation results, please see Tables 3, 4, 5.

**Table 3:** Correlation between average velocity of convergence and divergence and speed of rightward and leftward saccades at 80 ms and 160 ms for all participants.

		Rightward saccades at 80 ms	Rightward saccades at 160 ms	Leftward saccades at 80 ms	Leftward saccades at 160 ms
Average velocity during convergece	Pearson Correlation	− 0.19	− 0.21	− **0.22**	− **0.34**
	Sig. (2-tailed)	0.09	0.05	**0.04**	**0.002**
	N	84	84	**82**	**82**
Average Velocity during Divergence	Pearson Correlation	− 0.09	− 0.16	− **0.30**	− **0.40**
	Sig. (2-tailed)	0.41	0.16	**0.007**	**0**
	N	84	84	**82**	**82**

Variables with significant differences between the two populations (p < 0.05) are bolded.

**Table 4:** Correlation between average velocity of convergence and divergence and speed of rightward and leftward saccades at 80 ms and 160 ms for dyslexic participants.

		Rightward saccades at 80 ms	Rightward saccades at 160 ms	Leftward saccades at 80 ms	Leftward saccades at 160 ms
Average velocity during convergece	Pearson correlation	− 0.19	− 0.21	− 0.17	− 0**.36**
	Sig. (2-tailed)	0.25	0.19	0.32	**0.030**
	N	41	41	39	**39**
Average velocity during divergence	Pearson correlation	0.014	0.020	0.12	0.070
	Sig. (2-tailed)	0.93	0.90	0.48	0.66
	N	41	41	39	39

Variables with significant differences between the two populations (p < 0.05) are bolded.

**Table 5:** Correlation between average velocity of convergence and divergence and speed of rightward and leftward saccades at 80 ms and 160 ms for healthy participants.

		Rightward saccades at 80 ms	Rightward saccades at 160 ms	Leftward saccades at 80 ms	Leftward saccades at 160 ms
Average velocity during convergece	Pearson correlation	0.03	− 0.02	− 0.20	− 0.23
	Sig. (2-tailed)	0.86	0.91	0.20	0.13
	N	43	43	43	43
Average velocity during divergence	Pearson Correlation	0.03	− 0.08	0.06	− 0.06
	Sig. (2-tailed)	0.87	0.61	0.71	0.69
	N	43	43	43	43

Variables with significant differences between the two populations (p < 0.05) are bolded.

Finally, we examined correlations between subjective (stereotest, CISS scores, reading likeability) and objective results (vergence peak velocity and drift) for all children. We found that the stereotest score was negatively correlated with how much students reported they liked to read (r = − 0.3, p = 0.02). We also found that the stereotest was positively correlated with divergence duration (r = 0.4, p = 0.003), divergence peak velocity (r = 0.5, p = 0.001), and convergence peak velocity (r = 0.4, p = 0.01); the stereotest was negatively correlated with divergence average velocity (r = − 0.5, p = 0.001) and convergence average velocity (r = − 0.5, p = 0.001). Correlations between reading likability, CISS scores and objective measures did not reach significance. For a summary of these correlation results, see Table 6.

**Table 6:** Correlation between vergence parameters and subjective testing for all participants.

	Subjective Reading			Stereotest			CISS		
	Pearson correlation	Sig. (2-tailed)	N	Pearson correlation	Sig. (2-tailed)	N	Pearson correlation	Sig. (2-tailed)	N
Subjective reading	1		91	− 0**.32**	**0.02**	**91**	− 0.16	0.13	91
Divergence duration (ms)	− 0.001	0.99	90	**0.40**	**0.003**	**90**	0.053	0.62	90
Divergence peak velocity (°/s)	0.004	0.97	90	**0.45**	**0.001**	**90**	− 0.004	0.97	90
Divergence average velocity (°/s)	**0.21**	**0.049**	**90**	− **0.45**	**0.001**	**90**	− 0.037	0.73	90
Convergence peak velocity (°/s)	− 0.024	0.82	90	**0.36**	**0.01**	**90**	0.036	0.737	90
Convergence average velocity (°/s)	0.19	0.073	90	− **0.46**	**0.001**	**90**	− 0.13	0.22	90
Fixation disconjugacy 80 ms after convergence (°)	0.13	0.22	90	− 0.22	0.12	90	− 0.076	0.48	90
Fixation disconjugacy 160 ms after convergence (°)	0.076	0.47	90	− 0.2	0.16	90	− 0.076	0.48	90

Variables with significant differences between the two populations (p < 0.05) are bolded.

**Figure 1:**
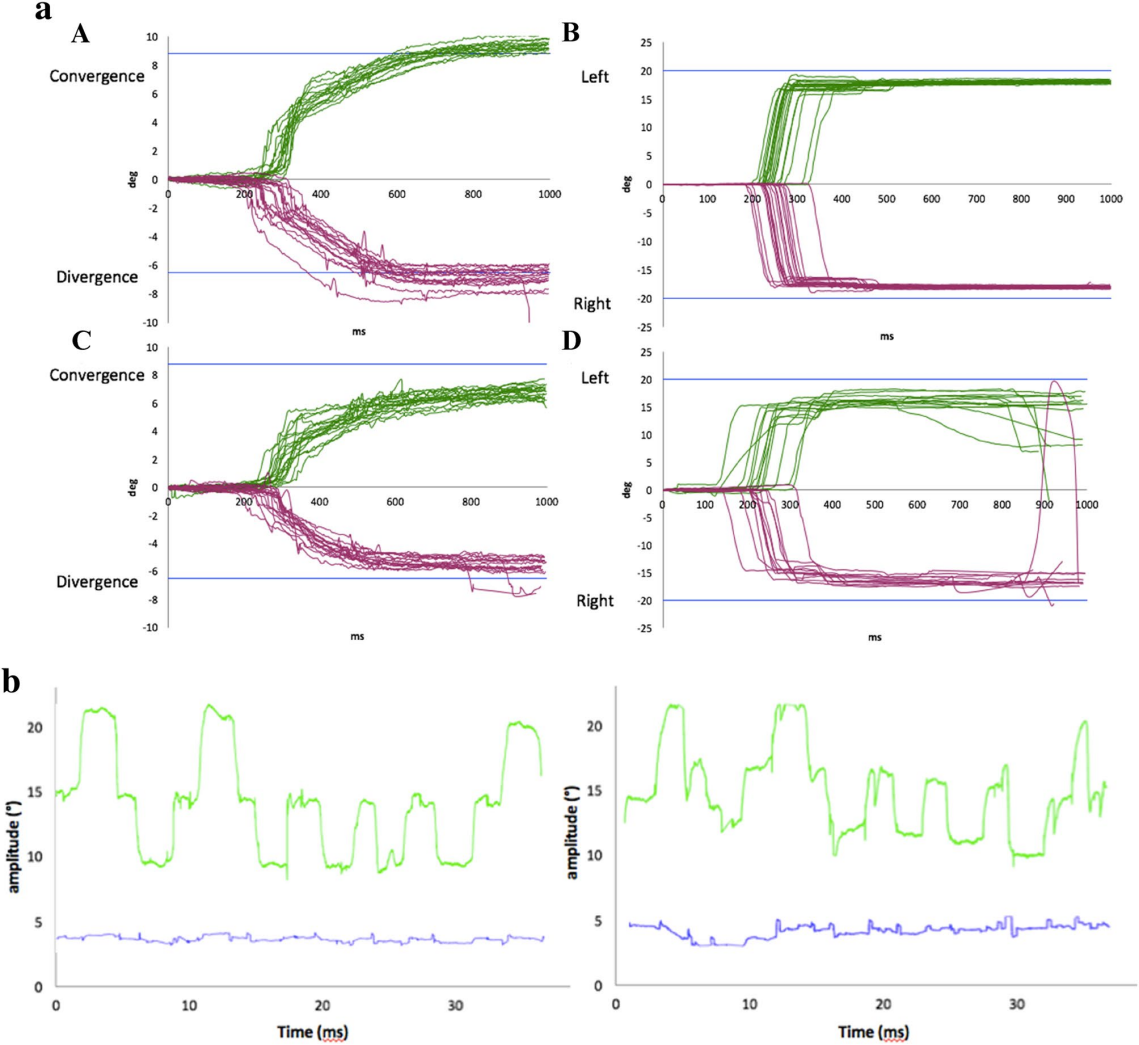
(**a**) Example of individual vergence and saccade trials. Tracings of vergence and saccades in an individual healthy control (**A**,**B**) and dyslexic (**C**,**D**) subject in one trial period. Despite initiating vergence movements quickly towards the target (in blue), the dyslexic subject takes longer to reach the vergence target (indeed, longer than measured). Similar difficulties can be seen in the saccade trial (**B**,**D**). (**b**) Example of vergence and conjugate tracing. Tracings of non-dyslexic (left) and dyslexic (right) vergence (green) movements over time. Convergence is represented by upward movements, divergence downward. The blue traces indicate conjugate saccade intrusions. Saccade intrusions are present for both, though are perhaps slightly more distinct for the dyslexic child; however, these measurements need finer analysis, perhaps with higher resolution eye trackers to better quantify eventual differences between the healthy control and the dyslexic.

An example of an individual dyslexic and non-dyslexic vergence and saccade tracings can be seen in Fig. 1a.

## Discussion

In this study, we demonstrate a novel method using REMOBI technology to objectively measure saccade and vergence disorders in dyslexics and non-dyslexics binocularly, without uncoupling vergence accommodation. Our study demonstrates subtle velocity abnormalities for both saccades and vergence and increased deconjugate post-saccadic drifts in dyslexics, suggesting inefficient binocular motor control in the dyslexic population. The tests used enable differential diagnosis of eye movement problems in dyslexia that could occur independently from reading: namely, that dyslexics’ reading difficulty could be partially a result of poor eye movement control.

### Vergence abnormalities

In terms of vergence, as compared to healthy adolescents, dyslexic adolescents show lower average velocities, suggesting that dyslexic adolescents have overall difficulty making prompt vergence movements, i.e. moving from near to far space and vice versa. This could have implications on their ability to rapidly and accurately perceive depth differences between objects in the three-dimensional space, including finding and focusing on words on a page. Psychophysical studies have established that depth perceptual ability relies on quality of vergence eye movements: divergence and convergence^39^.

Notably, this is the first time the profile of the vergence velocity was studied in the dyslexic population. Interestingly, dyslexic adolescents exhibited an abnormal velocity profile: they demonstrated a more robust, e.g. higher than normal, initial velocity (peak velocity) but their average velocity is slower; meaning their velocity is abnormally reduced in the later phases of movement for both convergence and divergence. From Hung’s model of double control of vergence, we can consider vergence to be modeled as a dual-mode control system^40^. The vergence response can be dissected into transient and sustaining components. The transient portion—the initial component—is assumed to be an open-loop control component of enhanced speed of a vergence response. The sustaining portion—the slow component—is assumed to be driven by a visual feedback, closed-loop control system. This closed-loop control system provides fine-tuning of the response and enables the extraordinary accuracy seen in binocular fixation.

Our observations are in line with Hung’s model as they demonstrate group differences in the initial and subsequent vergence components. The initial velocity movement during the transient, phasic phase in dyslexic adolescents is faster than that of healthy controls. However, the subsequent portion of the velocity including the sustaining portion (subsequent 160 ms), which is visually driven, is slowed. We postulate that this is due to delays in sensory visual processing in dyslexics. In previous studies we have reported a similar phenomenon in a healthy population, in which only the visually driven component of vergence is slowed as a result of age^41^. In the dyslexic population, this hypothesis of deficits in the visually-driven, sustaining portion of vergence is also consistent with previous theories regarding deficits in the magnocellular visual pathway that is responsible for processing dynamic, rapidly changing binocular disparity^17^.

There has also been previous literature that suggests that the feedback-controlled, or the slow component, of the vergence movement is affected by phoria, or the capacity to maintain the eyes aligned as one eye is covered. Indeed, previous studies have demonstrated that the phoria level is positively correlated with the vergence ratio (where convergence ratio = convergence peak velocity/divergence peak velocity). That is to say, the greater the increase in phoria, the greater the vergence ratio. As the eyes have a greater tendency to turn outwards away from each other (exophoria), they are more likely to have a convergence insufficiency leading to a decreased convergence peak velocity and a divergence excess, leading to an increased divergence peak velocity, ultimately giving a decreased vergence ratio. The opposite follows with esophoria, in which the eyes will either have a convergence excess and a more difficult divergence, leading to a higher vergence ratio^42^.

Could phoria be playing a role in dyslexics’ vergence velocity profiles? A previous study conducted by the lab with clinical orthoptic evaluation found no statistical difference in phoria between dyslexic and non-dyslexic populations; although heterophoria was more prevalent in the dyslexic population, it was only seen when measured at far distance^20^. Unfortunately, in the present study, phoria was not measured given our previous finding and the subjectivity of the test. We would encourage future studies in dyslexic populations to record phoria with objective eye movement measurements, as this could help explain the vergence discrepancies between the two populations. Finally, one could also consider that slowness in the sustained feedback system could also be attributed to a deficit in working memory given the classification of dyslexia as a primary learning disability that could involve memory deficits at least in some dyslexics. However, it is difficult to argue for such a mechanism delaying the execution of eye movements relative to healthy motor systems that produce movements at less than 500 ms.

In addition, it is important to consider the recruitment of saccades that may accompany vergence eye movements to better understand the differences velocity dynamics between the two populations. Generally, saccades occur with a faster velocity than vergence movements. Our own lab has demonstrated that horizontal saccades occurred in 84% of trials of vergence in healthy individuals^43^. Indeed, it has been previously demonstrated that the recruitment of additional oculomotor mechanisms, such as saccades, improved the phasic response properties of divergence in a healthy population, and that the prevalence of saccades is increased in vergence of eye movements as the vergence movement magnitude increases beyond 2°^44^. In this case, it could be that dyslexic adolescents had a higher prevalence of saccadic movements that accompanied the initial phase of vergence movements, giving them a higher initial peak velocity. Alternatively, if the higher peak velocities found in this study are related to an increase in the number of recruited saccades in the dyslexic population, one could infer a weaker vergence system in dyslexics that depends on recruited saccades to amplify the speed of the eye movement.

Indeed, on a review of a the vergence tracings, it appears that both the dyslexic and the non-dyslexic individuals do make saccadic movements within their vergence testing, perhaps of higher amplitude or more discrete in the dyslexic individual (Fig. 1b). Unfortunately, in the present study, it is impossible to extract data to quantify this aspect or further characterize these saccadic movements that accompany vergence movements. It can be attested that adolescents (either dyslexic or non-dyslexic), similar to healthy adults, do show saccade intrusions during their vergence eye movements. Future research should strive to further characterize these saccadic movements in dyslexic vs healthy populations.

Alternatively, abnormal velocity could be related to inappropriate tailoring of the pulse-slide-steps vergence motor command signals sent to extra-ocular muscles rather than to slower visual processing of the disparity and blur of retinal images^45^. In terms of motor control, the velocity of an movement depends on the quality of the movement generator signal located at the brainstem, i.e. in the mesencephalic reticular formation^46^. Inefficiency or dysfunction of the mesencephalic reticular formation in dyslexic adolescents could be at the origin of slower average vergence velocity. Whatever the reason, the impact of slower vergence is that clear single vision cannot be obtained as promptly as for non-dyslexics, i.e., dyslexic adolescents experience a delay in obtaining clear vision when moving their eyes from one depth to the other.

### Relation with clinical measures

It is notable that these vergence results, tested objectively with REMOBI lab equipment, correlate with some clinical evaluations we performed. Dyslexic adolescents tended to have lower scores of stereovision as tested by the Titmus test. They also had higher Convergence Insufficiency Symptom Survey results, indicating symptomatic vergence disorders. In order to further understand the relationship between clinical and objective testing, we performed correlation analysis between clinical testing and objective testing (see Table 6). The stereotest was significantly and negatively correlated with how much students reported they liked to read, which makes sense; we postulate that, as students have more difficulty with vergence and binocular coordination, reading becomes more difficult and they report liking to read less. In terms of correlations with objective measurements, we found that the stereotest was positively correlated with divergence duration. This is in keeping with our findings in comparing dyslexic vs healthy populations: as students have more trouble with coordinating binocular eye movements, they take longer to perform divergence; similarly, the same small vergence and saccade eye movements are stimulated during a stereovision test (e.g. the Titmus test we used here), leading to less efficient movements and therefore a lower stereovision capacity . It should be noted that the contribution of small eye movements in stereovision testing, to the best of our knowledge, has not been studied. The stereotest was also positively correlated with divergence and convergence peak velocity, suggesting that the higher the stereotest (or, the worse the stereovision capacity), the faster the initial vergence peak velocity. This is consistent with our findings: the dyslexic population studied demonstrated a higher peak velocity than healthy adolescents, which could be attributed to more saccadic recruitment during vergence movements, giving a more unstable vergence pattern as suggested above. Finally, the stereotest was negatively correlated with divergence and convergence average velocity, suggesting that the worse adolescents scored on the stereotest, the slower their average velocity during vergence movements. From these results, it seems that the stereotest, in particular, is a useful subjective, clinical examination related to vergence velocities. Overall these correlations between stereovision scores peak and average velocity seem to indicate that the quality of vergence profile can determine the stereovision scores. This is a novel field entirely unexplored area as sensory, visual, and motor performances are tightly linked. As our lab has argued in the past, subjective orthoptic testing can be used as preliminary testing, but it is important to objectively measure vergence, including during stereovision tests, in order to prescribe efficient rehabilitation^19^.

### Saccade abnormalities

In addition to vergence differences, dyslexics displayed differences in saccades in the left and right directions as compared to healthy controls. Notably, as in vergence, dyslexic adolescents demonstrated normal latency, but showed a faster initial peak velocity and a slower average velocity than non-dyslexics, leading to a longer total duration to reach their target. As saccade duration lasted on average less than 80 ms (see Table 2), and visual processing is approximately of the same duration, visual feedback is less likely to intervene in the control of the ongoing saccade, which is therefore executed under open loop control. In the case of saccades, this abnormality favors a hypothesis of motor inefficiency. It is possible that the slowing of saccades is due to inefficient vergence involvement leading to not only the subsequent deconjugate drift, but also the slowed saccade itself. If vergence control is lost during the initial part of the saccade, when the eyes are rapidly accelerating, a rapid vergence restoration mechanism is activated to reestablish this loss of vergence^13^. Yet, as we have shown vergence velocity is slowed in dyslexics, the saccade would in turn also be slowed, leaving larger vergence drifts during the subsequent fixation period. In fact, what we call disconjugate drift may be physiologically produced by the vergence system that is slowed in dyslexics and fails to restore vergence during the saccade itself. It has been previously shown that, while vergence normally accelerates the saccade, it can also slow it^47,48^. Vergence, or disconjugate drifts, during fixation harm visual acuity and therefore, single binocular vision^49^.

Interestingly, the difference in drifts was not statistically different after saccades to the left. Saccadic Left/Right asymmetries have been the subject of debate as previous studies have shown contradictory results^50–53^. However, most of these conflicting studies have been conducted in adults, rather than dyslexic adolescents. As rightward saccades are controlled by the contralateral ocular motor areas, involving visual, parietal, and frontal cortexes, perhaps the asymmetry in dyslexics could be attributed to left hemisphere dysfunction. Clinically, this finding could be related to dyslexic difficulty in fixating on words as they progress to the right along a line of text while reading. However, as previous studies on Chinese and Arabic speakers with dyslexia have contributed significantly to our understanding of the interplay between reading patterns and dyslexic pathology, futures studies should consider investigating these findings in dyslexics who read using non-Latin script^54–56^.

For the first time to our knowledge, this study indicates problems in saccade velocity and high deconjugate drifts following large amplitude saccades in the dyslexic population. While it has previously been shown that dyslexics have deconjugate post-saccadic drifts during reading, the present study suggests this problem is inherent, as it exists for large saccades tested objectively with the REMOBI device. The results favor the hypothesis previously proposed by our lab: that disconjugate drifts arise from poor vergence control^13,57^. Further, our lab has previously shown that experimental induction of vergence/accommodation mismatch negatively impacts disconjugate post-saccadic drifts^27^. Indeed, the present study argues that these vergence velocity abnormalities and disconjugate post-saccadic drifts are related.

### Correlation between vergence and saccade abnormalities

To substantiate this hypothesis regarding the physiologic link between vergence and saccades further, we examined the cross-correlation between average vergence velocity and saccadic deconjugate drifts (see Table 3). We found that the lower the velocity was during vergence, the higher the deconjugate drift of the saccades. This finding favors our hypothesis of a possible causal link between low vergence velocity capacity and poor binocular coordination after the saccade, or disconjugate drift.

When splitting the data of dyslexics and non-dyslexics, a significant negative correlation between average velocity and the disconjugate drift in the following 160 ms after the saccade was found for dyslexics only during convergence following left saccades (Table 4). These deficits in saccades and vergence confirm that the dyslexic adolescent population exhibits difficulty in both controlling vergence along the median plane and maintaining vergence stability during fixation after saccades (seen in the increased disconjugate drift).

### No latency abnormality in dyslexics

Contrary to previous studies in our lab, we did not find any latency abnormality in dyslexics for vergence or saccades. However, contrary to our previous experiments, in this study, we used the visual-acoustic REMOBI device, which provides a sound stimulus 50 ms before LED onset. Sound delivered in temporal proximity with the visual target reduces saccade latency; thus, in the present study, the normal latencies recorded for dyslexics may be mediated by sound acting as a warning stimulus, assisting dyslexics to trigger their saccades and vergence with normal latency^25^. Further research on dyslexics comparing eye movements to audio-visual vs visual targets are currently being conducted in our laboratory.

### Looking forward: lifestyle habits and rehabilitation

What else, outside of clinical and objective measures, could be associated with these eye movement abnormalities in the dyslexic population? Dyslexic adolescents tended to spend many more hours on the computer per week than non-dyslexic adolescents. It has been thought that dyslexic adolescents perform better when they are able to read and interact with a screen, so this finding may support their preference for screen learning^7–9^. Dyslexic adolescents also tended to like to go to theaters and films more than non-dyslexic adolescents, and, perhaps not surprisingly, liked reading less than non-dyslexic adolescents. We postulate that dyslexic adolescents also like to watch films more because they find reading too fatiguing. An open question that remains is to what extend prolonged used of computers aggravates the intrinsic vergence and binocular coordination problems of dyslexics.

As we have shown that dyslexic adolescents have poor eye movement in both vergence and saccades as compared to non-dyslexic adolescents, how can this new data inform the dyslexic community’s efforts to build rehabilitation and alternative learning programs for those affected?

Traditionally, dyslexic adolescents have been assigned to orthoptic rehabilitation, during which they perform exercises over many sessions designed to rehabilitate their eye movements. This helps many adolescents; however, of the 47 dyslexic adolescents studied, 34 had been to an orthoptist or were currently enrolled in orthoptic rehabilitation, yet continued to exhibit objective eye movement deficits and their clinical manifestations of those deficits. Are there any other methods that can be used to help them overcome their persistent deficits? Using computers instead of paper has been one strategy the dyslexic community has used to facilitate easier reading and writing in their population^7–9^. However, spending so much time reading on a screen can have some side effects: namely, increasing visual stress, which could accentuate reading problems^58–61^.

On the other hand, perhaps these preliminary results related to eye movement deficits could be useful in planning retraining exercises. Previous studies using a double step vergence paradigm on the REMOBI machine have shown lasting improvements in reading saccades, fixations, and vergence measurements^19,28,62–64^. Future research should repeat these experiments to verify audiovisual vergence retraining as a viable new rehabilitation technique. It may even be possible to use this method as a predictive test of dyslexic pathology using machine learning algorithms.

### Limitations

There are some limitations to this study. Although our population sampled is larger than most previous studies, the sample size is still relatively small, and we encourage future studies to confirm our findings. We were unable to quantify the severity of dyslexia in our population.

Yet, as recruitment was conducted at specialized schools for dyslexia, it is certain that these adolescents all had well documented, severe dyslexia to be admitted to this school, in which they were enrolled in special teaching classes for French and were followed by an orthophonist present in the school. As mentioned in the methods, the information shared by the school about the initial diagnosis of dyslexia run by a multidisciplinary neuropsychological center, indicated that 1/3 of the dyslexics had a visual type of dyslexia, another three a phonologic type, and the remainder were non-classified.

We were also unfortunately unable to quantify the directionality and amplitude of saccadic recruitment during vergence, which could have provided more valuable information regarding dyslexics’ abnormal velocity profile during vergence. Though we propose these abnormal eye movements, particularly those of vergence, may in fact be related to dyslexics’ difficulty reading, the present study does not provide evidence for the causality of the relationship. There have been no longitudinal studies or randomized control trials to determine if these abnormalities in eye movements are the cause of poor reading skill or a consequence of it. It would be useful for future research to follow children before starting and throughout their schooling to address this question, as it could help us better understand the fundamental problem in reading in the dyslexic population. Finally, although previous studies have shown that vergence and saccade eye movements improve with rehabilitation programs in patients with eye movement abnormalities attributed to other pathologies apart from dyslexia, future studies should also strive to determine if this improvement in eye movements is related to any potential improvements in reading skills. A pilot study in the lab suggests that 2 sessions of rehabilitation with the double step paradigm in depth on the REMOBI improves speed of reading by 20 words per minute^62,65^.

## Conclusion

Dyslexic adolescents exhibit slower vergence and saccades, with a velocity profile that shows a higher peak velocity in the beginning of the movement, but slow deceleration in the subsequent phase of movement towards targets in the three-dimensional space as compared to non-dyslexics. Though other studies have reported differences in saccadic and vergence eye movements in dyslexic adolescents, this study demonstrates an objective difference in the velocity profile of vergence and saccades in the dyslexic adolescent population. Slow average vergence velocity and disconjugate drifts following saccades can be causally related. Our study suggests dyslexics’ poor control of vergence dynamics alters saccade speed and induces disconjugate post-saccadic drifts. These deficits in both vergence and saccadic movement could provide an oculomotor basis for their reading difficulties: as it is more difficult to accurately and quickly locate the words in the three-dimensional space, it is more difficult to read.

Further, this study opens up the possibility for novel therapeutic rehabilitation techniques. The proposed testing method assesses purely motor problems independently from reading, which could become a useful clinical tool. Notably, the eye movement targets in this experiment mimic movements in everyday life: the targets were placed horizontally in the three-dimensional space at eye level and were preceded by a beep, which provides some warning and intersensory facilitation. This is an exciting area of research: though orthoptic training has been considered the standard of care rehabilitation for the dyslexic population, perhaps there are new avenues to design rehabilitation programs to train dyslexic adolescent eye movements to respond more quickly and accurately to these audiovisual stimuli. There have been some small preliminary studies that have already demonstrated improvement in reading (decreased regressive saccades, decreased fixation duration) in other populations^28,64^. In addition to the work that is currently being conducted, we would encourage more research in this exciting avenue specifically in the dyslexic population^65^.

Finally, and most importantly, we would like to highlight that reading is a multisensory activity that is fundamentally based around the visual system, of which eye movement is an integral component. Although we do not claim these eye movement problems represent the sole pathology in dyslexia, and although this study cannot claim to definitely declare eye movement pathology as a cause of poor reading ability, we believe the fragile nature of dyslexic adolescents’ eye movements could provide an inadequate basis on which to build efficient reading skills. These subtle eye movement deficits may represent a symptom of larger motor coordination disorders that may be associated with dyslexia and other learning disabilities. Therefore, we argue that these subtle eye movement testing should be considered as differential diagnostic criteria and used for evaluation and rehabilitation in the dyslexic population.

## Methods

### Participants

47 dyslexic adolescents (18 female, 29 male; mean age 15.4) and 44 non-dyslexic adolescents (22 female, 22 male; mean age 14.8) were recruited from schools in Paris. Dyslexic adolescents were diagnosed in specialized medical centers and were admitted to their school on the basis of their dyslexia diagnosis. These adolescents were all diagnosed in multidisciplinary centers where they underwent extensive multidisciplinary testing at the time of diagnosis, including neurological/psychological and phonological tests, as well as an evaluation of their speed of reading, comprehension, and capacity of reading words and pseudowords. In breaking down the types of dyslexia in the population, based on school records, 34.0% (16/47) identified their primary problem was visual/reading based, 4.3% (2/47) was auditory, 2.1% (1/47) was writing, and 59.6 (28/47) were mixed or unknown. It was impossible to separate the analysis out by primary dyslexic problem based on our small sample size. Other learning disabilities are a common comorbidity in dyslexic patients; of the dyslexic participants, twelve were also diagnosed with dysorthographia, dyscalcula, and/or dyspraxia. 34 had been to an orthoptist or were currently enrolled in orthoptic rehabilitation. Both dyslexic and non-dyslexic adolescents had no known neurologic or psychiatric abnormalities. Healthy controls had no known neurological or psychiatric abnormalities, no history of reading difficulty, no visual impairment, or difficulty with near vision. The investigation adhered to the principles of the Declaration of Helsinki and was approved by our Institutional Human Experimentation Committee (CPP CNRS 18 011). Written, informed consent was obtained from the adolescents and/or their parents after they were given an explanation about the experimental procedure. The tests were conducted by two research assistants, who were trained together using the same material and conducted the experiment together for each measurement.

### Clinical characteristics of the participants

Adolescents were asked to rate how much they liked doing certain activities (reading, watching movies, going to museums, etc.) on a scale of 1 to 10, where 1 indicated “not at all” and ten indicated “I love it”. They were also asked how many hours they spent watching TV, using the computer, playing video games, and on their telephone. They also responded to the Convergence Insufficiency Symptom Survey (CISS), a validated questionnaire for quantifying vergence problems in children and adults^33^. Their stereoscopic depth discrimination was measured using the Titmus Test (Titmus Stereo Fly Test, Stereo Optical, Essilor Instruments).

### Eye movement recording device

For each adolescent, eye movements were recorded binocularly with a head-mounted video-oculography device, Pupil Core, enabling binocular recording at 200 Hz per eye (Pupil Labs, Berlin).

### Calibration of the pupil labs device

The standard Pupil Labs calibration (Pupil Capture) was applied using a target that was presented at viewing distance of 1 m. The subject fixated on the center of the target and moved their head rightward, downward, leftward and upward at their own pace. They then repeated the sequence^66^.

### Ocular motor tests

Adolescents were asked to sit in front of a horizontal visual-acoustic REMOBI device. The device was placed at eye level, so that the first arc of LEDs was 20 cm from their eyes. Each child is instructed to carefully fixate quickly and accurately on the moving LED, to not attempt to predict a pattern of motion, and to keep their head still during the 2-min test.

**Figure 2:**
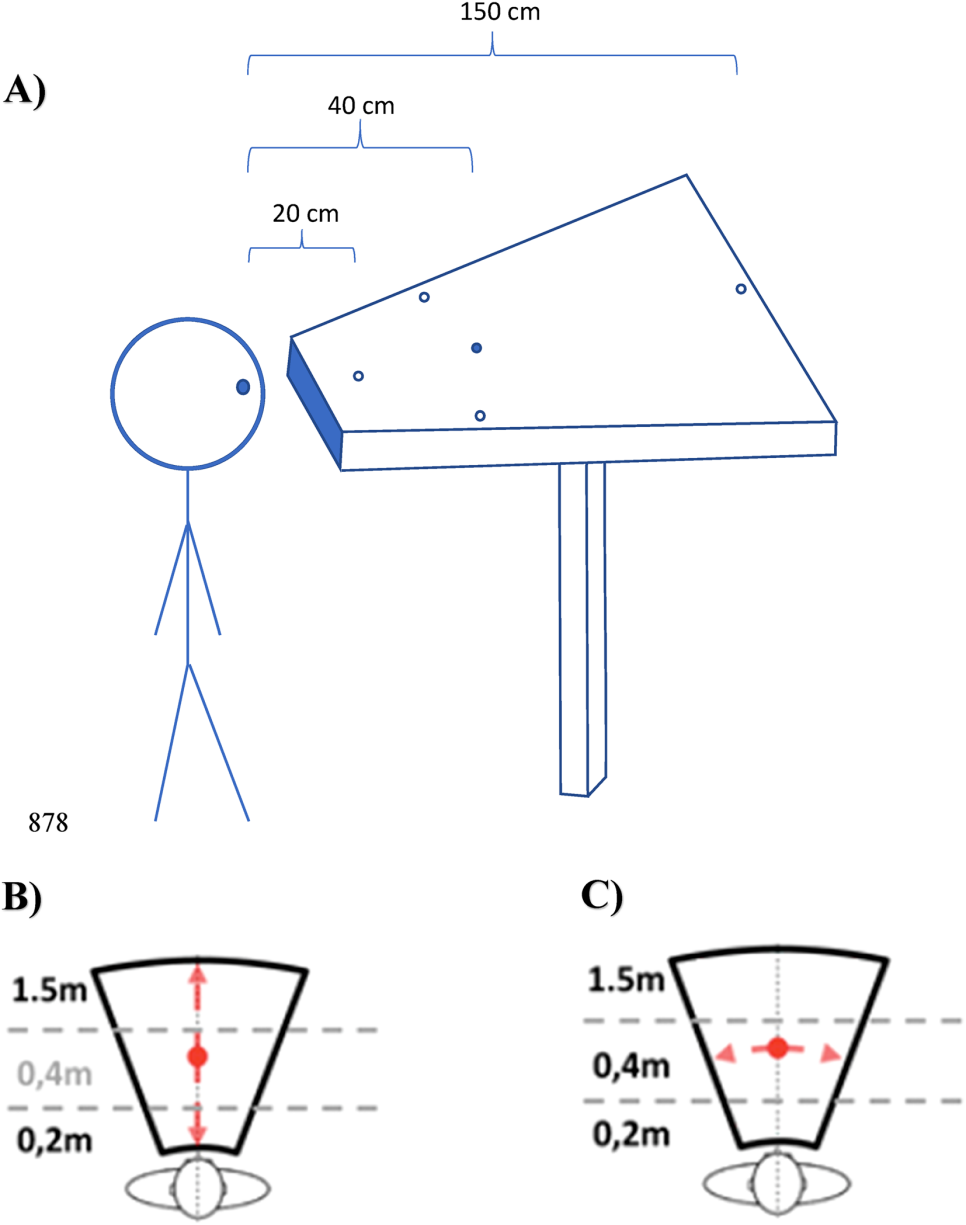
(**A**–**C**) Experiment set-up. Spatial (**A**) and temporal (**B**,**C**) arrangement of vergence and saccade tests. Subjects looked successively at different LEDs; from the initial (40 cm) fixation LED, to the target LED for divergence (150 cm) or the target LED for convergence (20 cm) (**B**); and from the initial (40 cm) fixation LED to the target LED for saccades (20° to left or right) (**C**). Each trial starts with the fixation target that appears for a variable period of 1200–1800 ms; following this period the target LED lights are on for 2000 ms together with a paired buzzer preceding 50 ms and lasting only 100 ms. (**C**) Arrows indicate the possible target locations for each test.

Oculomotor tests were performed in mesopic light conditions. Each test was conducted in a small room in the school that was quiet and free of distractions. The red LED stimuli were displayed at different distances, laterally or in depth, always in the horizontal plane (0°, Fig. 2A). LED characteristics were: nominal frequency 626 nm, intensity 180mCd, and diameter 3 mm. Adjacent to each LED was embedded a buzzer with the following characteristics: nominal frequency approximately 2048 Hz, sound pressure level 75 dB, diameter 12 mm.

### Vergence test

For each trial the fixation LED (0°) light up at 40 cm, creating a required vergence angle of 9° for a period varying from 1400 to 2000 ms (see Fig. 2B). It was followed randomly by the target LED during 2000 ms, appearing always at the central axis (0°) either at 20 cm (calling for a convergence movement of 8°, i.e. from 9° to 17°) or 150 cm (calling for a divergence movement of 7°, i.e. from 9° to 2). The test contained 40 trials (20 trials of convergence, 20 of divergence, pseudo-randomly interweaved) in an overlap paradigm: after an overlap period of 200 ms following the lighting of the target LED, the fixation LED switched off.

### Saccade test

Each trial started with the fixation central LED lighting at 40 cm from the subject for a randomized period ranging from 1400 to 2000 ms; it was followed by the lighting of the saccade target LED for 2000 ms at 20° of eccentricity, randomly chosen on the left or on the right (Fig. 2C). There were 40 trials (20 left, 20 right, pseudo-randomly interweaved) in an overlap paradigm.

### Data analysis

Data recorded with the Pupil Labs eye tracker were analyzed with AIDEAL, software developed in the IRIS laboratory. The vergence signal was derived by calculating the difference between the two eyes from the individual calibrated eye position signals (i.e., left eye–right eye). The beginning and end of the vergence movements were defined as the time point when the eye velocity exceeded or dropped below 5°/s: these criteria are standard and were applied automatically by the AIDEAL software; the program estimated the initial phasic component as the amplitude between these two initial points. It also calculated the amplitude change during the subsequent 80 ms and 160 ms. The total amplitude was calculated as the sum of the amplitude of the initial phasic component plus the 160 ms component. The total duration was calculated as the duration of the phasic component plus the subsequent 160 ms.

For saccade analysis, AIDEAL treated the conjugate signal, e.g. the L + R eye position/2. The onset and the offset of the saccade were defined as the time points where the peak velocity went above or below 10% of the peak velocity; practically, this corresponded to values above or below 40°/s (as the peak velocity of 20° saccades is typically above 40°/s). The total average velocity was defined as the ratio of total amplitude in degrees divided by time in seconds. To evaluate binocular coordination of saccades, or the disconjugacy during saccadic movements, the difference in amplitude between the left and the right eye signal was calculated. The disconjugate drift, or the difference in drift amplitude during the first 80 or 160 ms of fixation, was calculated. These calculations are standard and have been used in previous experiments^62,63^.

Trials with blinks or other artifact were discarded automatically by AIDEAL. For each adolescent (dyslexic and non-dyslexic) the number of saccades and vergence movements measured in the fixation tasks were counted. The percentage of movements rejected were 0% for vergence and 9% for saccades in dyslexic children and 2% for vergence and 3% for saccades in healthy children.

### Statistical analysis

As the measured eye movements data were not normally distributed as determined by the Shapiro–Wilk test, the non-parametric Mann–Whitney U test was utilized for means comparison. All hypothesis testing was two-sided and p-values of ≤ 0.05 was considered statistically significant.

In a second analysis, we calculated the Pearson correlation between the average velocity during divergence and convergence and the drift during leftward and rightward saccades at 60 and 180 ms. We calculated the Pearson correlation for all subjects, for dyslexic subjects only, and for healthy subjects only.

In a third analysis, we calculated the Pearson correlation between the Stereotest scores, the CISS scores, and the objective vergence data (Duration, Peak Velocity, Average Velocity and Fixation Disconjugacy at 80 and 160msec in convergence and divergence). We calculated this score for all subjects, dyslexic subjects only, and healthy controls only.

For all analyses, the statistical significance was set at p ≤ 0.05. All analyses were performed using SPSS version 25 (IBM Corp. Released 2017. IBM SPSS Statistics for Windows, Version 25.0. Armonk, NY: IBM Corp.).

## Data Availability

The datasets generated during and/or analyzed during the current study are available from the corresponding author on reasonable request.
